# Prevalence of social disconnection in the general adult population – results from DISCONNECT study

**DOI:** 10.1007/s00127-026-03093-5

**Published:** 2026-04-29

**Authors:** Michal Hajdúk, Valentína Cviková, Sára Majsniarová, Jakub Januška, Alexandra Straková, Vladimír Ivančík, Natália Čavojská, Adam Kurilla, Daniel Dančík, Hana H. Kutlíková, Anton Heretik, Petra Brandoburová, Simona Krakovská, Marianna Mrva, Daniel Gerbery, Roman Džambazovič

**Affiliations:** 1 Department of Psychology, Faculty of Arts, Comenius University in Bratislava, Bratislava, 811 04 Slovakia; 2 Department of Psychiatry, Faculty of Medicine, Comenius University , Bratislava, Slovakia; 3 Institute for Sociology of the Slovak Academy of Sciences, Bratislava, Slovakia; 4 Department of Sociology, Faculty of Arts, Comenius University , Bratislava, Slovakia

**Keywords:** Loneliness, Social isolation, Social disconnection, Mental health

## Abstract

**Purpose:**

Social disconnection, consisting of loneliness and social isolation, is increasingly recognized as a significant public health problem. This cross-sectional population study aims to investigate the prevalence of social disconnection in the general adult population of the Slovak Republic and its associations to socio – demographic/economic and health indicators.

**Methods:**

A representative sample of 3006 adults from the Slovak Republic was recruited for this study. Data were collected through standardized questionnaires via face-to-face interviews during November 2024. Chi-square tests and logistic regressions were used to examine the associations between covariates and social disconnection.

**Results:**

The prevalence of social disconnection was found to be 25.3% for loneliness and 19.1% for social isolation. Both lonely and isolated were about 9% of the population. Significant associations were identified between social disconnection and mental health conditions, with higher levels of loneliness and social isolation linked to increased severity of depression. Several subpopulations showed higher risks of social disconnection.

**Conclusion:**

This study highlights the pervasive nature of social disconnection in the Slovak Republic and its substantial impact on both mental and somatic health. Our findings underscore the need for comprehensive national health policies focusing on social disconnection especially in at-risk populations such as elderly women with lower educational attainment, discriminated individuals, individuals in financial strain, and parents on maternity/paternity leave.

**Supplementary Information:**

The online version contains supplementary material available at https://doi.org/10.1007/s00127-026-03093-5.

## Introduction

Building and maintaining close and meaningful relationships constitute a fundamental human motivation force [1]. Despite extensive evidence supporting this need, many societies experience decreasing levels of social connection and social engagement [2, 3]. In response, the World Health Organization [4], European Commission [5], and U.S. Surgeon General [6] have identified loneliness and social isolation as one of the main public health problem, with global significance.

Loneliness is usually defined as a subjective experience of insufficient quality or quantity of social relationships. In contrast, social isolation refers to an objective condition describing an individual’s actual frequency of social contacts [7, 8]. When studied together, these constructs are often characterized using the umbrella term social disconnection. The latest report of WHO Commission on Social Connection [4] highlights that social disconnection is widespread in all regions of the World and all age groups. While their effects are partly overlapping, loneliness and social isolation represent distinct risk factors, Loneliness affects nearly one in six people globally. The most extensive meta-analysis of the prevalence of loneliness in 113 countries showed that loneliness affects a significant part of the population across countries. However, the included studies showed considerable heterogeneity in prevalence estimates, which was substantially driven by different measures and various cut-offs that can bias direct comparisons [10]. According to the recent European Union data from a large-scale international survey conducted in 2022 with over 25 000 respondents, approximately 13% of people reported feeling lonely most of the time or all the time [10]. The prevalence of loneliness was lowest in Austria, Croatia, Czechia, the Netherlands, Slovenia and Spain (10% or less). The highest rates were observed in Bulgaria, Cyprus, Ireland and Luxembourg (17% or more). Differences across countries may reflect differences in culture and values but also differences in the demographic composition of the national population as well as sampling differences [11].

Data from three pooled European Social Survey waves (2010, 2012, and 2014, *N* > 106000) revealed that 18% of European adults were socially isolated [12] Social isolation seems to follow clear regional patterns. Western and Southern Europe have the lowest levels of socially isolated individuals (approx. 14,5%), with Eastern Europe at the other extreme (33,4%) [13]. In addition, a European Social Survey round 9 indicated that less than 2% of people aged 15 and older are severely socially isolated [14]. Regional differences are underscoring how the broader socio-cultural and socio-economic country context may exert an impact on individual circumstances [5, 15].

Age consistently appears as a primary demographic risk factor associated with social disconnection. Based on recent meta-analytic estimates, it was shown that about 1 in 3 older adults experience loneliness or social isolation [16–19]. Furthermore, a large analysis of 9 longitudinal studies from different countries showed a U-curve shaped association between loneliness and age. Adolescents and young adults, alongside older adults, show the highest risk for loneliness [20]. These results might suggest different developmental trajectories for loneliness and social isolation, since younger age is typically associated with rich social life and social isolation is reported as a risk factor for loneliness specifically in older adults [21].

The results on sex differences are mixed. A meta-analytic study has found negligible differences (g = 0.07) in loneliness between males and females, with age being the only significant moderator of sex differences [22]. Another research suggests the sex differences in social isolation may be moderated by the interaction between age and partnership status [23]. Specifically, males appear to be more isolated across their lifespan, especially if they do not have stable relationships [24]. Living with others and being married is, on average, linked to a lower loneliness risk [25]. However, there is an evidence of the shift in population trends, such as divorced individuals tend to report a lower level of loneliness than cohorts of divorcees reported in previous decades [26].

In terms of other demographic factors, educational attainment was not consistently associated with social disconnection, especially in multivariate models [25]. UK Biobank data indicate that individuals with lower socio-economic status and income reported higher loneliness and social isolation, with a stronger association in males [27]. Loneliness and social isolation also show some urban-rural variations. For example, according to the UK Biobank data, individuals living in the most crowded areas were more likely to feel lonely and socially isolated than those in the least crowded areas [28]. In Germany, loneliness was unequally distributed across the country, and these differences go beyond traditional urban-rural distinction [29].

Decades of research have shown that loneliness and social isolation are connected to various physical and mental health outcomes [30]. Social disconnection is prospectively linked to increased morbidity and mortality for oncological and cardiovascular diseases [31]. There is also evidence that social isolation is linked to cognitive decline in older age [32, 33] and type II diabetes [34]. Having close relationships has proven to be protective for health, as it is linked to a 50% decrease in the likelihood of mortality [35]. Higher levels of loneliness and social isolation are also present in individuals with mental illness, with exceptionally high prevalence linked to depression [36] and psychotic disorders [37, 38]. The mechanisms underlying associations between social disconnection and health are complex. It has been proposed that social disconnection causes severe social stress that leads to the activation of the HPA (hypothalamic-pituitary-adrenal) axis. Chronic disconnection in particular can lead to dysregulation of immune response and elevated inflammation, resulting in various health problems [7, 39].

The objectives of this study are threefold. First, to estimate the prevalence of social disconnection, specifically loneliness and social isolation, in the general adult population (18 years and older) of the Slovak Republic. Second, to compare levels of social disconnection across key demographic and socio-economic groups (age, sex, educational attainment, partnership status, employment, income, subjective socio-economic status). Finally, the study examines the association between social disconnection and health status – both mental and physical.

## Methods

### Study design

DISCONNECT is a population-based longitudinal study designed to investigate the prevalence of social disconnection in the Slovak adult population. The data were collected between October and November 2024, with participants at home in the presence of an interviewer. The procedure involved a combination of face-to-face interviews. and self-administered questionnaires completed on tablets (Computer assisted personal interviews - CAPI). The questionnaires were administered in two languages, Slovak and Hungarian. The mean duration of the interview was 45 min. In total, 200 trained interviewers collected data from 3006 individuals. each completing a maximum of 30 interviews. To minimize potential interviewer effects, all interviewers underwent standardized training, and the total workload was distributed such that each interviewer completed a maximum of 30 interviews. Furthermore, the risk of interviewer bias was expected to be minimal because all key study measures were based on direct self-report assessment. The total adult population of the Slovak Republic at the time of data collection was approximately 4 393 876.

A two-stage stratified sampling approach was used. In the first step, primary sampling units – i.e., municipalities (in the case of Bratislava and Košice, urban districts) – were selected based on random stratified sampling. The stratification criteria were the region (8 regions) and, within the region, the size of the municipality by population (7 size categories - up to 999, 1 000–1 999, 2 000–4 999, 5 000–19 999, 20 000–49 999, 50 000–99 999, 100 000+). The number of municipalities for the combination of county and settlement size category was selected in proportion to the number of adult residents living in that municipality size category. The number of primary sampling units was 300. Quota characteristics were sex (2 categories), age (6 categories), education (4 categories), and nationality (3 categories). The quotas corresponded to the distribution of the adult population at the county level. Only one respondent was interviewed per household.

Inclusion criteria were adults aged 18 years or older, residing in the Slovak Republic during the study period, and able to provide informed consent. Exclusion criteria were institutionalization, cognitive impairment preventing consent, or other conditions precluding completion of the survey (e.g., language barriers). All participants provided informed consent, and the study was approved by the Ethical Board of the Faculty of Arts, Comenius University in Bratislava (ER/32A/2024).

## Measures

### Socio-demographic and socio-economic indicators

Sex was categorized as male or female. Age was analyzed as a continuous variable, except for the sample description, which used age categories. Education was divided into three categories, and household size was measured by the number of people living in the household, including the participant. Income was classified into nine bands, and partnership status was grouped into the following categories: no partner, married, divorced, or widowed. Employment status was classified into eight categories. Urban-rural classification had four categories based on settlement size. In addition, subjective social status was measured as a self-reported position in the hierarchy within the society using MacArthur scale of Subjective Social Status, a single item with a 10-point scale where higher numbers indicate higher perceived status [40].

## Social Disconnection

Social disconnection (loneliness and social isolation) was assessed using direct single-item questions and standardized psychometric instruments. Using two direct questions, we asked participants: *How often have you felt lonely/isolated during the last four weeks?”* Response options were *never*, *hardly ever*, *sometimes*, *often*,* and always*. These questions were drawn from the EU Loneliness survey [10]. Furthermore, loneliness was measured using the 3-item version of the UCLA Loneliness Scale [41]. This scale assesses subjective feelings of loneliness by asking participants how often they feel they lack companionship, feel left out, or feel isolated from others. Responses are recorded on a 3-point Likert scale (1 = hardly ever, 2 = sometimes, 3 = often). The total score ranges from 3 to 9, with higher scores indicating greater loneliness. In accordance with the cut-offs applied in the large-scale EU Loneliness Survey (EU-LS 2022), participants scoring 6 and above were categorized as lonely, and those scoring 8 or 9 were classified as very lonely. The scale demonstrated good internal consistency in our sample (Cronbach’s α = 0.846).

Social isolation was assessed using the Lubben Social Network Scale (LSNS-6) [42], which evaluates the social network size and the frequency of social contact. The instrument consists of six items, with three items assessing family networks and three assessing friendship networks (e.g., “How many relatives/friends do you see or hear from at least once a month?“). Responses are scored on a 6-point scale (ranging from 0 = none to 5 = nine or more). The total score ranges from 0 to 30. Following the empirical cut-off recommended by the scale authors [42], scores below 12 were used to indicate social isolation. The internal consistency for the LSNS-6 in the current study was good (Cronbach’s α = 0.856).

## Subjective health

Subjective somatic and psychological health were each assessed with a single Likert-type item. Participants were asked to rate their overall physical and mental health using a five-point scale: 1 - very bad, 2 - bad, 3 - neither good nor bad, 4 - good, 5 - very good.

### Depression

Depressive symptoms were measured using the Patient Health Questionnaire-9 (PHQ-9) [43]. The PHQ-9 includes nine items assessing the frequency of depressive symptoms (e.g., ‘Little interest or pleasure in doing things’) over the past two weeks, with higher total scores indicating greater symptom severity. Participants rate each item on a 4-point Likert scale ranging from 0 (not at all) to 3 (nearly every day). The total score ranges from 0 to 27, with higher scores indicating greater depressive symptom severity. The PHQ-9 showed good internal consistency in our sample (Cronbach’s α = 0.882).

### Statistical analysis

The analysis plan was pre-registered on the Open Science Framework (https://osf.io/hvr2z). The prevalence of social disconnection was estimated for the total sample and across socio-demographic and socio-economic strata. Differences across stratification variables were examined via chi-square tests or Mann-Whitney U/t-tests, depending on the nature of the data or distribution of scores. In addition, we analyzed how social disconnection (measured with direct questions) is related to self-reported perception of mental and physical health. We calculated the proportions of socially disconnected people per response category.

In addition to bivariate analysis, we used logistic regression to examine the independent effects of covariates on social disconnection. Predictors included age, sex, education attainment, number of people in the household, partner status, employment, urbanicity, subjective socio-economic status, being diagnosed with mental illness, and current depression severity. In some variables (education, employment) we collapsed categories to simplify model. The strength of associations was presented as odds ratios with 95% CI. In models predicting loneliness, a dependent variable was the binary outcome, non-lonely vs. lonely, based on the UCLA − 3, with the higher cutoff (8 and above). This will be our primary interest model for loneliness. Another model with the less strict cutoff for loneliness (6 and above) was computed to check for the robustness of the findings. Same set of predictors was used in the model for social isolation (LSNS-6) as a binary variable with the cutoff at 12 points. To evaluate and, where necessary, correct for minor deviations between the achieved sample and the Slovak adult population, post-stratification weights were calculated based on joint distributions of key sociodemographic variables. Specifically, weights were derived for the following variable combinations: sex × age, age × education, sex × settlement size, age × settlement size, education × settlement size, region × settlement size, and nationality × region. Population benchmarks were obtained from annual updates published by the Statistical Office of the Slovak Republic.

Given that the final sample closely matched population characteristics across the key sociodemographic strata, the main regression analyses were conducted without stratification weights. To evaluate the robustness of the results, weighted regression models were additionally estimated as sensitivity analyses; these are reported in the Supplementary Material and yielded essentially similar findings. All analyses were performed using SPSS v30.

## Results

### Characteristics of the sample

A total of 3006 participants were included in the analysis. The mean age of respondents was 48.51 years (SD = 16.45). Women represented 52.5% of the sample. With respect to education, 12.3% of participants completed primary education, 65.7% secondary education, and 22.0% tertiary education. Detailed information about the characteristics of the sample is displayed in Table 1..

**Table 1.** Characteristics of the sample

	%
Sex	
Male	47.5
Female	52.5
Age	
18–24	8.6
25–34	15.4
35–44	19.7
45–54	18.4
55–64	15.8
65 and more	22.1
Education	
Elementary	12.3
Secondary	65.7
University	22.0
Nationality	
Slovak	89.3
Hungarian	7.9
Other	2.8
Urban	
Large city	25.1
Suburbs of large city	4.7
Medium or small town	28.0
Village	42.3
Marital status	
Single	29.1
Married	50.0
Divorced	12.1
Widower	8.7
Perceived income adequacy	
Can live comfortably	23.4
Can handle it	49.9
Having a hard time	21.6
Having a very hard time	5.2

### Prevalence of social disconnection

Overall, the prevalence of loneliness in the study population was 25.3%, while 19.1% of participants reported being socially isolated. When combined, 9% experienced both loneliness and social isolation simultaneously. When we applied stricter criteria for loneliness, 5.2% of participants were classified as very lonely. Furthermore, 34.8% of lonely individuals were also socially isolated, while 46% of socially isolated individuals were lonely. The prevalence values did not substantially differ with applied weights. Participants reported varying levels of loneliness and social isolation during the past four weeks (direct questions). Regarding loneliness, 46.0% indicated they were never lonely, 25.8% reported hardly ever, 21.2% sometimes, 5.9% often, and 1.1% always. Similarly, for social isolation, 59.4% reported never feeling isolated, 19.2% hardly ever, 16.0% sometimes, 4.7% often, and 0.7% always.

Demographic differences in loneliness and social isolation are presented in Table 2. Women reported higher levels of loneliness compared to men (28.1% vs. 22.3%). Older adults were more likely to experience loneliness (36.7%) and social isolation (24.5%) than all other age groups. Individuals with elementary educational attainment showed the highest prevalence of both loneliness (37.9%) and isolation (26.3%). Lastly, participants living in large urban areas reported greater levels of loneliness than those living in smaller municipalities (30.4%).

**Table 2:** Demographic characteristics and social disconnection

		Lonely		Socially isolated	
		%	Sig. difference^†^	%	Sig. difference^†^
Sex	Male	22.3	0.07***	19.3	0.01
	Female	28.1		18.8	
Age	18–24	17.7	0.15***	9.2	0.11***
	25–34	21.9		14.1	
	35–44	20.3		18.3	
	45–54	23.1		20.0	
	55–64	25.9		21.5	
	65 and more	36.7		24.5	
Education	Elementary	37.9	0.11***	26.3	0.07***
	Secondary	24.0		18.2	
	University	22.4		17.7	
Urban	Large city	25.1	0.961	20.3	0.273
	Suburbs of large city	25.7		13.6	
	Medium or small town	24.9		18.3	
	Village	25.8		19.4	
Marital status	Single	24.7	0.20***	19.0	0.10***
	Married	19.3		16.0	
	Divorced	35.1		26.3	
	Widower/Widower	48.5		27.1	
Perceived income adequacy	Can live comfortably	15.4	0.21***	13.7	0.14***
	Can handle it	23.0		17.5	
	Having a hard time	35.3		24.5	
	Having a very hard time	51.3		35.9	
Household type	One-person household	44.6	0.22***	28.6	0.13***
	Childless couple	21.8		18.3	
	One-parent household	25.9		24.4	
	Two-parent household	17.0		14.2	
	Intergenerational household	26.7		15.8	
	Other	24.2		16.8	
Employment	Employed	18.3	0.22***	15.1	0.13***
	Unemployed	41.7		25.2	
	Student	20.6		10.6	
	Probation period	43.8		25.0	
	On a disability pension/long-term sick leave	57.0		43.0	
	Retired (on an old-age pension)	36.5		25.2	
	Homemaker (not on maternity/parental leave)	34.3		34.3	
	Other (maternity leave, caretaker)	11.8		35.3	
Feel discriminated	No	22.7	0.08***	18.0	0,08***
	Yes	49.8		29.1	
Diagnosed with mental disorder	No	23.0	0.22***	17.6	0.16***
	Yes	64.7		44.1	

^†^Values represent Cramer’s V from Chi-squared tests of independence; *p <.05; **p <.005; ***p <.001

### Association between loneliness and self-rated health

Substantial differences in perceived mental health were observed by loneliness and social isolation (Table 3). Among not lonely individuals, a substantially higher proportion reported very good or good mental health as compared to lonely individuals, who were more likely to rate their mental health as bad, or very bad. A similar pattern was found for social isolation, with non-isolated individuals more often reporting very good or good mental health, while socially isolated individuals shifted toward poorer ratings. Both associations were statistically significant (loneliness: χ² = 590.11, *p* <.001, V = 0.44; social isolation: χ² = 174.42, *p* <.001, V = 0.24). The results were similar for perceived physical health. Lonely and socially isolated participants were more likely to rate their physical health as negative. Chi-square tests again indicated significant associations for both loneliness (χ² = 367.65, *p* <.001, V = 0.35) and social isolation (χ² = 129.64, *p* <.001, V = 0.21). The exact percentages are displayed in Table 3.

**Table 3:** Social disconnection and perceived health

		Lonely %		Socially isolated %	
		No	Yes	No	Yes
Mental health	Very bad	0.05	0.7	0.1	0.5
	Bad	0.8	8.9	1.6	7.9
	Neither good nor bad	9.9	37.1	13.9	29.3
	Good	50.6	46.9	50.8	44.7
	Very Good	38.6	6.4	33.5	17.6
Physical health	Very bad	0.4	1.8	0.5	1.9
	Bad	4.5	19.8	6.5	16.4
	Neither good nor bad	24.3	41.5	26.6	37.3
	Good	48.2	31.8	46.3	34.6
	Very Good	22.7	5.1	20.2	9.8

### Logistic regression analysis

Three logistic regression models were estimated. The results are presented in Table 4. For loneliness models, Nagelkerke’s R^2^ were 0.310 and 0.319. For social isolation model, Nagelkerke’s R^2^ was 0.126. Several independent risk factors emerged. In case of stricter cut-off for loneliness, the significant predictors were being married (OR = 0.32, 95% CI[0.18, 0.56]), unemployment due to long-term health problems (OR = 0.2.36, 95% CI[1.16, 4.82]), subjective status (OR = 0.74, 95% CI[0.67, 0.83]), treatment for mental illness (OR = 2.16, 95% CI[1.31, 3.55]), and depression severity (OR = 1.20, 95% CI[1.16, 1.24]). The odds of loneliness were significantly increased for living in a smaller household (OR = 0.89, 95% CI[0.81, 0.98]), being unemployed (OR = 1.59, 95% CI [1.08, 2.34]) or on disability (OR = 1.70, 95% CI[1.01, 2.87]), lower subjective status (OR = 0.84, 95% CI [0.79, 0.89]), treatment for mental illness (OR = 2.17, 95% CI [1.47, 3.21]), and depression severity (OR = 1.21, 95% CI [1.19, 1.24]). In contrast, social isolation was predicted by household size, employment, treatment for mental illness, and depression severity. A similar pattern to loneliness was observed for social isolation, except subjective status was not a significant predictor. All reference categories for categorical predictors are marked in Table 4.

**Table 4:** Logistic regression models for social disconnection

	Lonely strict (UCLA-3 − 8–9)					Lonely (UCLA-3 > 6)					Social isolation < 12				
	B	Sig.	OR	95% C.I.		B	Sig.	OR	95% C.I.		B	Sig.	OR	95% C.I.	
**Age**	0.003	0.756	1.003	0.982	1.026	0.001	0.922	1.001	0.989	1.012	0.009	0.100	1.009	0.998	1.021
**Sex**	0.003	0.987	1.003	0.682	1.475	0.075	0.459	1.078	0.884	1.315	− 0.193	0.058	0.824	0.675	1.006
**Education – elementary – reference**															
Secondary	− 0.131	0.604	0.877	0.534	1.441	− 0.266	0.073	0.766	0.573	1.025	− 0.215	0.150	0.806	0.601	1.081
University level	0.518	0.111	1.679	0.888	3.174	− 0.108	0.558	0.898	0.626	1.288	− 0.141	0.446	0.868	0.604	1.248
**Household size (no. of people)**	− 0.102	0.262	0.903	0.756	1.079	− 0.118	0.013	**0.888**	0.809	0.976	− 0.196	< 0.001	**0.822**	0.744	0.908
**Marital status – single – reference**															
Married	−1.144	< 0.001	**0.319**	0.180	0.564	− 0.400	0.007	0.671	0.501	0.897	− 0.334	0.025	0.716	0.535	0.958
Divorced	− 0.510	0.107	0.600	0.323	1.117	0.083	0.642	1.087	0.766	1.542	− 0.027	0.877	0.973	0.689	1.374
Widower	− 0.021	0.954	0.980	0.490	1.960	0.410	0.057	1.507	0.988	2.300	− 0.242	0.266	0.785	0.513	1.202
**Employment – employed – reference**															
Unemployed	− 0.184	0.627	0.832	0.396	1.747	0.463	0.019	**1.589**	1.080	2.338	0.431	0.030	**1.539**	1.042	2.272
Student	− 0.224	0.707	0.799	0.248	2.576	0.038	0.890	1.039	0.608	1.775	− 0.278	0.376	0.757	0.409	1.403
Disability – long-term health problems	0.858	0.019	**2.359**	1.155	4.818	0.533	0.045	**1.704**	1.011	2.870	0.577	0.021	**1.781**	1.092	2.906
Retired	0.569	0.087	1.766	0.921	3.388	0.305	0.074	1.357	0.971	1.897	0.090	0.593	1.095	0.786	1.525
**Urban – large city – reference**															
Suburb of large city	− 0.031	0.953	0.969	0.340	2.760	0.270	0.269	1.310	0.812	2.115	− 0.412	0.133	0.662	0.387	1.134
Medium or small town	0.304	0.246	1.355	0.811	2.264	0.073	0.591	1.076	0.824	1.404	− 0.135	0.318	0.874	0.670	1.139
Village	0.379	0.116	1.462	0.910	2.347	0.199	0.109	1.221	0.957	1.557	0.039	0.752	1.039	0.818	1.321
**Subjective SES**	− 0.292	< 0.001	**0.747**	0.668	0.836	− 0.174	< 0.001	**0.840**	0.790	0.893	− 0.059	0.056	0.943	0.888	1.002
**Treatment for mental illness**	0.769	0.002	**2.157**	1.312	3.546	0.777	< 0.001	**2.174**	1.472	3.212	0.711	< 0.001	**2.036**	1.421	2.919
**Depression – PHQ-9**	0.178	< 0.001	**1.195**	1.156	1.236	0.191	< 0.001	**1.211**	1.185	1.237	0.077	< 0.001	**1.080**	1.059	1.102
**Nagelkerke R2**	0.310					0.319						0.126			

B = unstandardized regression coefficient; Sig. = significance level (p-value) testing whether B significantly differs from zero; OR = odds ratio; 95% C.I. = 95% confidence interval for the odds ratio

## Discussion

The goal of the study was to estimate prevalence of loneliness and social isolation in the Slovak adult population. We looked at the prevalences stratified for relevant social, demographic, and economic factors as well as link to mental and somatic health. We found that approximately one quarter of the adult population in the Slovak Republic reported experiencing loneliness (25,3%), and 19,1% were socially isolated. In addition, one in ten adults experienced both loneliness and social isolation simultaneously. Taking together, a substantial part of Slovak adult population lacks proper social connections. Our results showed similar prevalence to those estimated in previous meta-analyses [10, 19, 44]. Importantly, our findings highlight that loneliness and social isolation are related but distinct constructs. We found that only 34.8% of lonely individuals were also objectively socially isolated. This indicates that the majority of lonely adults (approximately 65%) maintain standard social networks yet still subjectively feel a lack of meaningful connection. Conversely, more than half (precisely 54%) of socially isolated individuals were not lonely. This suggests that for many individuals, a small social network may be a personal preference or a circumstance to which they have comfortably adapted. These findings emphasize the necessity of measuring both constructs separately. Consequently, interventions targeting network expansion may not effectively alleviate subjective loneliness if the quality of relationships is the underlying issue.

We found that socio-demographical and socio-economic factors were linked to increased levels of loneliness and isolation. While our initial analyses indicated that older adults, women, and individuals with lower educational attainment experienced higher prevalences of social disconnection, these effects were attenuated in the fully adjusted regression models. Reported odds ratios indicate that the primary independent risk factors are partnership status, economic stability, and mental health. Possible explaining mechanisms include lack of social support, partner loss, and reduced overall opportunities for social engagement. Economic deprivation and financial limitations [45] also emerged as an important risk factor that is not limited to older population or deprived neighborhoods [46], highlighting the need for policies that actively support employment and provide adequate social protection, including supported living arrangements for those at risk. Our results confirmed that poor financial circumstances (insufficient income) and unemployment are significant risk factors for loneliness and social isolation. A specific group warranting attention are adults on maternity or paternity leave. We found that they reported higher levels of loneliness despite not being isolated. This finding suggests that people may experience strong feelings of loneliness even when surrounded by loved ones. High demands of childcare, the inability to meet personal needs during upbringing, lack of support or empathy from others may contribute to emotional distress and, consequently, loneliness [47–49]. Potential ways to improve social connectedness in this group include flexible employment arrangements, mental health programs and interventions targeting parents, and opportunities for community building through interactions with other parents. The final group at elevated risk of social disconnection was individuals who perceived discrimination. This was most common among those with non-heterosexual orientation and among individuals identifying as members of other marginalized groups. There may be common mechanisms in these subgroups (such as internalized stigma, and reduced access to supportive networks) that are likely related to perceived social exclusion. Interventions addressing social exclusion [50], network or community building [51, 52] may positively influence perceived social connectedness. Overall, the estimated regression models for loneliness and social isolation showed largely similar patterns, supporting their overlap as distinct, but related dimensions of social disconnection. Together, our results may sugges that loneliness is more closely tied to how people perceive their social position and relationship quality, whereas social isolation is more strongly linked to structural disconnection from social and occupational roles. The lower loneliness among married individuals further supports the view that close relational bonds may be more protective against subjective disconnection than against objective isolation.

We examined how social isolation and loneliness were associated with both somatic and mental health. Our results confirmed well-established associations, showing that both factors play an important role in health outcomes at the population level. In addition, both loneliness and social isolation were associated with the severity of depression. Several mechanisms may account for these findings, including heightened stress responses, dysregulation of inflammatory and neuroendocrine pathways [39]. From ong-term perspective, it might add to increased morbidity and mortality [30, 53]. In the Slovak Republic, approximately 70–80% of individuals with depression and anxiety disorders do not receive appropriate treatment [54]. This treatment gap may increase the risk of social disconnection. At the same time, socially disconnected individuals face greater barriers to receive appropriate care. Targeted efforts focusing on increasing social connections could therefore play an important role in reducing gap and improving population mental health. Policies that focus on community, personal and relational factors, and stigma reduction therefore help break the vicious circle of mental illness and social disconnection [4]. Our previous analyses [55] indicated that non-comprehensive local or regional strategies currently exist to address social disconnection in the Slovak republic. Our findings therefore provide strong empirical evidence to support further development of such initiatives.

The results need to be interpreted considering several limitations. First, we used the three-item loneliness scale, which is a well-established tool for large-scale surveys. However, due to its lower reliability, it may lead to the misclassification of some individuals as lonely. Despite this, another measure of loneliness showed analogous patterns. Second, although this was a population-based study, institutionalized older individuals were not included in the survey. This is important, as this subpopulation is known to experience particularly high levels of loneliness [56]. Third, the cross-sectional nature of the present analysis does not allow us to infer causality between social disconnection and health outcomes, because exposures and outcomes were measured at the same time. We are currently preparing the second wave of the project, which will address this limitation. Fourth, because we adhered to our preregistered analytic plan, our regression models may yield conservative estimates due to potential overadjustment if some included covariates act as mediators between predictors and outcomes. Fifth, reliance on self-reported data introduces the potential for memory and social desirability biases, which might lead to some underreporting symptoms. These risks were partially mitigated by the study design, which used self-administered questionnaires on tablets (CAPI) to capture sensitive experiences, and included standardized measures with short recall periods (e.g., the past two to four weeks). Finally, although the sample is representative of the general population in the Slovak Republic, self-selection bias may still have occurred.

## Conclusion

In this study, approximately one quarter of the adult population in the Slovak Republic experience loneliness, and nearly a fifth was found to be socially isolated. In addition, one in ten adults experience both loneliness and social isolation simultaneously, reflecting a substantial burden of social disconnection in the Slovak adult population. Social disconnection was significantly associated with poorer mental and physical health outcomes. Our findings underscore the need for comprehensive national health policies focusing on social disconnection especially in at-risk populations such as elderly women with lower educational attainment, discriminated individuals, individuals in financial strain, and parents on maternity/paternity leave.

## Supplementary Information

Below is the link to the electronic supplementary material.


Supplementary Material 1


## Data Availability

The data used in the current study is available from the corresponding author on a reasonable request.
