# Supplementary material for: Prevalence of social disconnection in the general adult population – results from DISCONNECT study
Source: Soc Psychiatry Psychiatr Epidemiol. 2026 Apr 29;61(8):1401–11. doi: 10.1007/s00127-026-03093-5 (PMC13522021; doi:10.1007/s00127-026-03093-5)
Supplement: Supplementary file 1 [file 127_2026_3093_MOESM1_ESM.pdf]

Prevalence of social disconnection in the general adult population – results from DISCONNECT study

Supplementary file

Logistic regression models with weighted data are presented in the following tables. Same set of predictors as in the main part or manuscript were used.

Table 1. Lonely strict (UCLA - 3 - 8-9)

	B	S.E.	p	OR	95% C.I.	
**Age**	.000	.011	.975	1.000	.979	1.022
**Sex**	-.076	.198	.702	.927	.629	1.366
**Education – elementary – reference**						
Secondary	-.109	.255	.668	.897	.544	1.477
University level	.408	.323	.207	1.504	.798	2.833
**Household size (no. of people)**	-.109	.087	.213	.897	.756	1.065
**Marital status – single – reference**						
Married	-1.255	.288	<.001	.285	.162	.501
Divorced	-.439	.312	.159	.644	.349	1.188
Widower	-.110	.348	.751	.895	.453	1.772
**Employment – employed – reference**			.100			
Unemployed	-.165	.374	.659	.848	.407	1.765
Student	-.636	.656	.332	.529	.146	1.913
Disability – long-term health problems	.777	.364	.033	2.174	1.065	4.441
Retired	.627	.334	.061	1.871	.972	3.603
**Urban – large city – reference**						
Suburb of large city	.037	.550	.947	1.037	.353	3.049
Medium or small town	.310	.261	.236	1.363	.817	2.276
Village	.410	.244	.093	1.506	.933	2.431
**Subjective SES**	-.292	.058	<.001	.747	.667	.836
**Treatment for mental illness**	.712	.258	.006	2.037	1.228	3.381
**Depression – PHQ-9**	.177	.017	<.001	1.194	1.155	1.235

B = unstandardized regression coefficient; Sig. = significance level (p-value) testing whether B significantly differs from zero; OR = odds ratio; 95% C.I. = 95% confidence interval for the odds ratio

Table 2 Lonely (UCLA-3 > 6)

	B	S.E.	p	OR	95% C.I.	
**Age**	.000	.006	.953	1.000	.989	1.012
**Sex**	.044	.102	.668	1.045	.856	1.276
**Education – elementary – reference**						
Secondary	-.169	.148	.254	.845	.632	1.129
University level	.000	.180	.999	1.000	.703	1.423
**Household size (no. of people)**	-.102	.047	.031	.903	.823	.991
**Marital status – single – reference**						
Married	-.425	.147	.004	.654	.490	.871
Divorced	.033	.181	.856	1.033	.725	1.472
Widower	.397	.212	.061	1.488	.981	2.255
**Employment – employed – reference**						
Unemployed	.435	.197	.027	1.545	1.051	2.270
Student	.041	.273	.882	1.041	.610	1.777
Disability – long-term health problems	.472	.264	.074	1.603	.955	2.691
Retired	.290	.171	.089	1.337	.957	1.869
**Urban – large city – reference**						
Suburb of large city	.394	.244	.106	1.483	.919	2.391
Medium or small town	.143	.137	.297	1.153	.882	1.507
Village	.248	.127	.050	1.282	1.000	1.643
**Subjective SES**	-.192	.031	<.001	.826	.776	.878
**Treatment for mental illness**	.636	.198	.001	1.889	1.280	2.787
**Depression – PHQ-9**	.195	.011	<.001	1.216	1.189	1.243

B = unstandardized regression coefficient; Sig. = significance level (p-value) testing whether B significantly differs from zero; OR = odds ratio; 95% C.I. = 95% confidence interval for the odds ratio

Table 3 Social isolation - LSNS < 12

	B	S.E.	p	OR	95% C.I.	
**Age**	.005	.006	.337	1.005	.994	1.017
**Sex**	-.181	.102	.076	.834	.683	1.019
**Education – elementary – reference**						
Secondary	-.214	.149	.150	.807	.603	1.080
University level	-.168	.181	.352	.845	.593	1.205
**Household size (no. of people)**	-.224	.051	<.001	.799	.723	.883
**Marital status – single – reference**						
Married	-.213	.147	.149	.808	.606	1.079
Divorced	.065	.178	.717	1.067	.752	1.513
Widower	-.162	.215	.451	.850	.558	1.296
**Employment – employed – reference**						
Unemployed	.413	.201	.040	1.511	1.020	2.238
Student	-.222	.309	.473	.801	.437	1.468
Disability – long-term health problems	.687	.247	.005	1.988	1.224	3.228
Retired	.141	.169	.404	1.151	.827	1.602
**Urban – large city – reference**						
Suburb of large city	-.601	.297	.043	.548	.306	.982
Medium or small town	-.089	.135	.509	.915	.702	1.192
Village	.065	.124	.599	1.067	.837	1.361
**Subjective SES**	-.057	.031	.066	.944	.888	1.004
**Treatment for mental illness**	.587	.187	.002	1.799	1.247	2.594
**Depression – PHQ-9**	.078	.010	<.001	1.081	1.060	1.104
**Age**	-1.040	.381	.006	.353		

B = unstandardized regression coefficient; Sig. = significance level (p-value) testing whether B significantly differs from zero; OR = odds ratio; 95% C.I. = 95% confidence interval for the odds ratio
